# Excimer laser ablation combined with drug-coated balloon versus drug-coated balloon in the treatment of de novo atherosclerotic lesions in lower extremities (ELABORATE): study protocol for a real-world clinical trial

**DOI:** 10.1186/s12872-022-02751-1

**Published:** 2022-07-16

**Authors:** Xiaolang Jiang, Longhua Fan, Bin Chen, Junhao Jiang, Jianjun Liu, Guanyu Qiao, Shuai Ju, Yun Shi, Tao Ma, Changpo Lin, Gang Fang, Daqiao Guo, Xin Xu, Zhihui Dong, Weiguo Fu

**Affiliations:** 1 Department of Vascular Surgery, Institute of Vascular Surgery, Zhongshan Hospital, Fudan University, National Clinical Research Center for Interventional Medicine, 180 Fenglin Road, Shanghai, 200032 China; 2 Department of Vascular Surgery, Qingpu Branch of Zhongshan Hospital, Fudan University, 1158 East Gongyuan Road, Shanghai, 201715 China; 3 Department of Vascular and Wound Treatment Center, Jinshan Hospital, Fudan University, Shanghai, 200540 China

**Keywords:** Excimer laser ablation, Drug-coated balloon, De novo lesions, Atherosclerotic obliterans

## Abstract

**Background:**

The efficacy and validity of excimer laser ablation (ELA) in the in-stent restenosis (ISR) has been confirmed. However, its application in de novo atherosclerotic lesions of lower extremity artery disease (LEAD) has not been clearly defined and its procedure has not been standardized.

**Methods:**

ELABORATE is a prospective, multicenter, real-world study designed to evaluate the efficacy and safety between ELA combined with drug-coated balloon (DCB) and DCB alone in de novo atherosclerotic lesions of LEAD.

**Discussion:**

ELABORATE is a prospective, multicenter, real-world study designed to assess the efficacy and safety between ELA combined with drug-coated balloon (DCB) and DCB alone in patients with de novo atherosclerotic lesions of LEAD. According to the real-world situation, eligible patients will be allocated to ELA + DCB group (group E) and DCB group (group C). Baseline and follow-up information (at 3, 6, and 12 months) will be collected. The primary efficacy point is primary patency at 12-months, and the secondary efficacy points include clinically driven target lesion reintervention (CD-TLR), change of Rutherford class, ankle-brachial index and ulcer healing rate. These indexes will be assessed and recorded at 3, 6, and 12-month follow-up. Also, safety evaluation, including major adverse event, all-cause mortality through 30-day follow-up, unplanned major amputation, bailout stent and distal embolization, will also be evaluated by an independent core laboratory. All the data will be collected and recorded by the electric data capture system. This study will be finished in 3 years and the 12-month results will be available in 2023. All the patients will be followed for 5 years.

*Trial registration number* Chinese Clinical Trial Registry (ChiCTR2100051263). Registered 17 September 2019. http://www.chictr.org.cn/listbycreater.aspx.

## Background

With the increase of aging population, the incidence rate of peripheral artery disease (PAD) is getting higher [1]. In Germany, the prevalence of PAD has reached 10% [2]. The prevalence is up to 20% among 70-year-old German patient population [1]. More than 20 million people worldwide were diagnosed with PAD in the year of 2009 [3, 4]. And it cost 4 billion in the treatment of PAD patients per year [5]. Of note is the fact that the symptoms are not classic in the early stage in many patients. However, approximately 10–15% of them would progress to critical limb ischemia (CLI) within 1 year [6]. Meanwhile, they are also more likely to suffer from other cardiovascular diseases [7]. Hence, the aim of treating patients with PAD is to improve the long-term results and to improve the quality of life (QoL) and thus to reduce the national medical burden.

Lower extremity artery disease (LEAD) is the most commonly involved location in patients with PAD. In the past decades, endovascular therapy has become the main method to treat these patients. Meanwhile, more centers prefer the endovascular approach compared with open surgery. Even in patients with long and complex LEAD, endovascular therapy also demonstrates a relatively high technical success and low risks. Currently, endovascular therapy mainly included percutaneous transluminal angioplasty (PTA) and stent implantation; however, the drawbacks of these procedures are increasingly appeared. Studies have reported that the vessel patency was significantly higher in stents compared to PTA alone when the length of femoropopliteal lesions was < 5 cm [8]. The implantation of bare-mental stent could prevent the occurrence of extensive recoil and dissection thus to improve clinical outcomes in long segment lesions. However, in-stent restenosis (ISR) with an incidence rate of 14–50%, is the main factor affecting the long-term clinical outcomes [9–12]. In order to prevent ISR, drug-eluting stents has been applied recently, and no significant difference has been identified compared with bare-mental stents regarding freedom from restenosis at 24-months [13].

The superiority of drug-coated balloon (DCB) in de novo atherosclerotic lesions and ISR has been demonstrated in previous studies [14–17]. However, flow-limiting dissection and recoil are still observed up to 20% of de novo lesion and 16% of ISR after the application of DCB, which is needed to be treated by stenting [16–18]. Furthermore, calcification in the intima or media could interfere with the absorption and distribution of paclitaxel and reduce the effect of DCB, which has been proven to be an independent risk factor of DCB [19]. In addition, the patency of stent implantation in non-stent zones is poor due to the special anatomy. The incidence rate of ISR at 3-year reaches 45.8% [20]. Meanwhile, the clinical outcome of stents implantation in below-the-knee (BTK) arteries is unclear. It is well known that patients with CLI mainly involved BTK arteries, especially in those with diabetes mellitus or chronic kidney dysfunction [21]. Also the efficacy of PTA in such patients need to be clearly defined, especially given the high incidence of re-stenosis due to the diffused calcification and plaque [22]. Excimer laser ablation (ELA) could minimize the dissection compared to PTA without debulking, owing to removal of plaque and calcification. Its efficacy in ISR lesions has been demonstrated and also been approved by U.S. Food and Drug Administration (FDA) [23]. However, its efficacy in de novo atherosclerotic lesions has not been clearly defined. ELABORATE is initiated as the first trial to compare the safety and efficacy of ELA combined with DCB in patients with de novo atherosclerotic obliterans (ASO) in a real-world setting.

## Methods

### Trial design

**Fig. 1:**
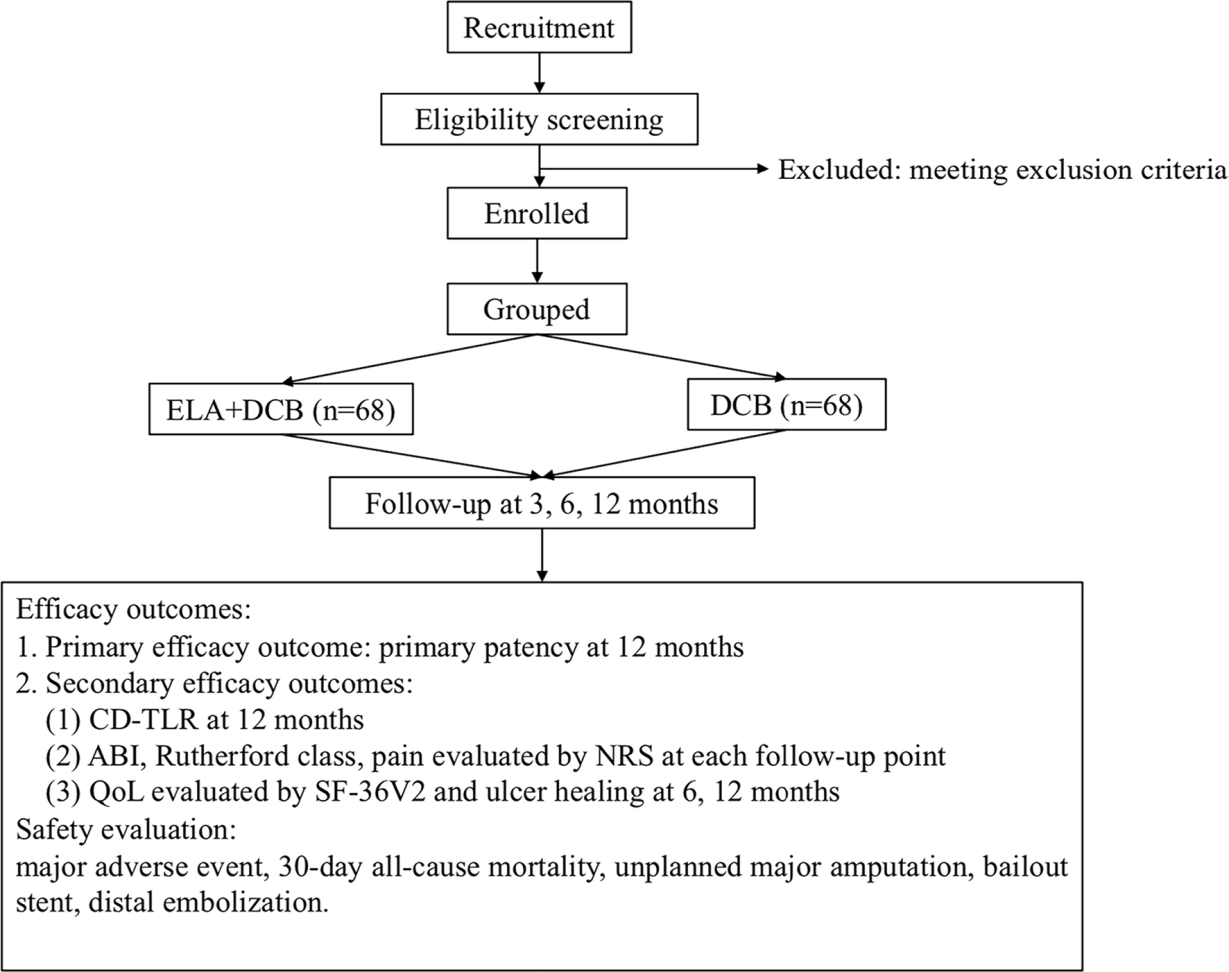
The flow-chart of the ELABORATE trial

This trial is a prospective, multicenter, real-world study, and it has been approved by the Ethics Committee of Zhongshan Hospital, Fudan University (Approval number: B2021-578). The overall design is demonstrated in Fig. 1. The data collection is shown in Table 1. The design of this trial is accordance with the Standard Protocol Items [24].

**Table 1:** Schedule of enrollment and follow-up points

			Follow-up visits		Long-term follow-up	Measuring
Outcome variables	Baseline	Intervention	1 month	3, 6, 12 months	(Every 12 months until 60 months)	Instrument
Allowed visit window	4 weeks to 1 week		± 5 days	± 10 days	± 30 days	
	Before intervention					
Informed consent	X					
Inclusion and exclusion criteria	X					
Calcification class		X				PACSS
Target lesion length		X				
Location of lesions		X				
Runoff vessels		X				
Minimal luminal diameter (a)		X				DSA
Reference vessel diameter (b)		X				DSA
Minimal luminal diameter (c)		X				IVUS
Reference vessel diameter (d)		X				IVUS
ABI		X		X	X	
Rutherford class		X		X	X	
Ulcer size		X		X	X	
Pain		X		X	X	NRS
QoL		X		X	X	SF-36V2
Primary patency				X	X	DUS
All-cause mortality			X			
Major amputation				X	X	
Bailout stent		X				
Distal embolization		X				
Study completion/termination record	At study exit					

*ABI* ankle-brachial index, *QoL* quality of life, *PACSS* peripheral arterial calcium scoring system [25], *DSA* digital substract angiography, *IVUS* intravascular ultrasound, *NRS* numerical rating scale, *DUS* Doppler ultrasound

(a) (b): evaluated by DSA in both groups before and after intervention; (c) (d): evaluated by IVUS in study group before and after laser

### Objectives

The primary objective is to demonstrate whether the application of ELA in the de novo LEAD is associated with an increased rate of efficacy outcomes.

### Recruitment

This study will be carried out from September 1, 2021 to 30 June, 2024. Each participant will be fully notified by the trial leaders from each center and signs the informed consent.

Inclusion criteria.

The inclusion criteria of this trial are as follows:

1. Aged ≥ 18 years.
2. Target lesion length ≤ 10 cm of the femoropopliteal and BTK arteries.
3. Significant stenosis ≥ 70% or chronic total occlusion (CTO) lesions assessed by the angiography.
4. Rutherford class 2–5 with ankle-brachial index (ABI) < 0.9 in the target limb.

Exclusion criteria are as follows:

1. ISR lesions.
2. Acute limb ischemia.
3. Simultaneously usage of other debulking devices.
4. Serum creatinine > 250 umol/L unless dialysis-dependent, contrast agent allergy, coagulation dysfunction, and other severe comorbidities that is impossible to tolerate the procedure.

### Interventions

The establishment of access was as our previous study [25]. And the extend of the calcification of the targeted lesions will be assessed according to Peripheral Arterial Calcium Scoring System (PACSS) [26]. The length, runoff vessels situations, minimal luminal diameter (MLD), and the reference vessel diameter (RVD) will be visually assessed under the digital subtraction angiography (DSA).

After passage of the lesion, the necessity of the usage of the distal protective device is at the surgeon’s discretion. One runoff vessel existed and contaminant (1) long-segment CTO or ISR lesions; (2) severe calcified lesions; (3) subacute thrombosis, is recommended the application of the distal protective device. For patients in the treatment group, the laser guide (Spectranetics Corp., Colorado Springs, Colorado) will be used to achieve maximum debulking with starting parameter: 45 mJ/mm^2^, 45 Hz, followed by 50–60 mJ/mm^2^ and 50–70 Hz. The angiography will be performed to evaluate the MLD, distal embolization, the extend of dissection. ELA could be continued if the lumen acquired is not satisfactory.

After ELA in the treatment group, the following procedure is similar in two groups. A standard balloon, 0.5-mm undersized to the RVD, is used before the DCB. And DCB is selected with 1:1 ratio to RVD for 2 min. Angiography is performed at least two different angles to record the MLD and dissection (if existed). In the treatment group, intravascular ultrasound (IVUS) (Volcano Corporation, Rancho Cordova, CA, USA) is applied to confirm the type of the lesions, MLD, and RVD before ELA, and MLD, RVD, and extend of dissection after ELA. The use of other debulking devices is prohibited. The bailout stent is implanted if flow-limiting dissection > 40% or residual stenosis > 30% occurs. The distal protective device would be retrieved and the debris > 2 mm will be recorded significant ones [27].

Any special balloon, such as Chocolate balloon, cutting balloon or Shockwave balloon, will be prohibited.

### Follow-up strategy

These patients will be given aspirin (100 mg/d) and clopidogrel (75 mg/d) for at least 6 months after intervention. And they will be followed at 3, 6 12 months. ABI and Doppler ultrasound of the targeted lesions will be evaluated at each follow-up point, and SF-36QoF is used to assess the QoL. The pain score during the follow-up is evaluated by the numerical rating scale (NRS). Meanwhile, the ulcers (if existed) will be photographed at two different angles. All participants will be followed for at least 5 years.

Participant can withdrawal from the trial any time, and their routine treatment would not be affected. The allocated interventions can be modified when (1) participant request, (2) the allocated intervention does no good to participant’s disease. Dr Zhihui Dong is responsible for any changes.

### Outcomes

#### Efficacy outcomes

The primary efficacy outcome is primary patency at 12-month, which is defined as no clinically driven target lesion reintervention (CD-TLR) and peak systolic velocity ratio (PSVR) ≤ 2.5 at the target lesion assessed by duplex ultrasound. The secondary efficacy outcomes are: (1) CD-TLR, (2) technical success, defined as residual stenosis ≤ 30% and flow-limiting dissection ≤ 40%, (3) ABI, (4) change of Rutherford class, (5) pain evaluated by NRS, (6) QoL evaluated by SF-36V2, and (7) ulcer healing.

Safety evaluation includes major adverse event (MAE), all-cause mortality through 30-day follow-up, unplanned major amputation, bailout stent, and distal embolization.

The definition of adverse event (AE) and adverse device effect (ADE) are according to Zeller et al. [28]. Any AEs and ADE will be recorded.

### Sample size

A minimum sample size of 136 patients is determined to be required for this study, assuming 1-sided α = 0.025, β = 80%, assumed 76.1% and 84.9% primary patency at 12 months in the DCB and ELA + PTA groups, respectively, and 25% attrition due to the nature of the real-world study [23, 29].

### Statistical analysis

Data will be analyzed by SPSS 20.0 (IBM Co., Armonk, NY, USA). The distribution of continuous variables will be checked firstly. Continuous variables were presented as mean ± standard deviation (SD) with range values for normally distributed data, or as the medians with the interquartile ranges (IQRs) for non-normally distributed data, respectively. Categorical variables were presented as frequencies and percentages. The differences between continuous variables are identified by using 2-sided Student t tests or the Wilcoxon rank test. While chi-square test or Fisher exact test is applied to compare the difference between categorical variables. All of the tests are 2-sided, and a *p* value < 0.05 was considered statistically significant.

### Assignment of interventions

According to the real-world situation, the group participants will be allocated completely depends on the patients’ request. Hence the sequence generation is not applicable. And the double-blind method is also impossible in this trial.

### Data collection, monitoring and confidentiality

Each patient’s ID and baseline information will be collected. The occurrence and timing of clinical outcomes will be documented. The ABI and measurements of ulcer size will be recorded and performed by one special technician using the same apparatuses. Participant retention and follow-up engagement is enhanced by communicating verbally and via WeChat app. A case report form (CRF) is used to collect data for each participant. Meanwhile, the collected data will be stored in an electronic data capture (EDC) system to ensure data authenticity.

The strategy of monitoring will be carried in the middle and at the end of the trial just as Chen et al. [30]. No audit is planned for the study. Meanwhile, the measures that used to keep the confidentiality and safety of data are consistent with those took by Chen et al. [30]. And the paper copies of the CRFs will be kept by the trial leaders for at least 5 years.

The results of this trial will be present at vascular conferences or be published in vascular journals. Data can be provided to researchers upon request, subject to a review of privacy. Requests for data can be sent to the investigator, Zhihui Dong, by email.

### Composition, roles, and responsibilities of the coordinating center, steering committee

The steering committee, including the principal investigator Zhihui Dong, has full responsibility for the design and implementation of this trial. Local primary investigators at each site are responsible for study administration, coordination, and monitoring of this trial at their own centers.

### Ethics approval

This trial is approved by Ethics Community of Zhongshan Hospital, Fudan University (B2021-578) on August 30, 2021. Also it has been registered in the Chinese Clinical Trial (ChiCTR2100051263). Reassessment will be made by ethics communities once there is any changes of the plan.

## Discussion

Guideline on treatment of ASO by ELA are primarily based on retrospective studies or randomized controlled trials (RCT) [23, 31]. However, retrospective study complies with its heterogeneity nature and RCT does not represent the real clinical practice due to its registration purpose. Only a small proportion of patients are eligible for RCT due to the strict inclusion and exclusion criteria [23].

ELABORATE is the first trial comparing the efficacy of ELA combined with DCB and DCB alone in the treatment of de novo atherosclerotic lesions in a real-world setting. Compared with RCT, real-world study more conforms to clinical practice and provides high external validity [32]. The inclusion criteria in ELABORATE represent the broad definition of a patient eligible for endovascular therapy in the real world. Meanwhile, study accessibility is maximized by defining minimal exclusion criteria. Observational studies and RCTs all have their own nature limitations and any of them alone may not fully represent the true value of certain treatment for LEAD [33]. As such, well-designed real-world study may offer complementary data to these standard types of studies, representing true real-world effectiveness. The application of ELA in ISR has been approved by U.S. FDA [23]. And it also has been approved in our nation in the treatment of PAD. However, its validity in PAD, especially in de novo atherosclerotic lesions, has not been fully demonstrated, and the operation is still not standardized.

Although been approved by Chinese FDA, performing a trial of relatively new device in real-world setting still poses many challenges. Participant’s safety is the priority of the study. We have several steps to keep the enrolled participant’s safety. Firstly, before initiation, surgeons who participates in the trial will be trained by professional engineer about the principles of excimer laser, also they will be trained by an experienced vascular surgeon who performed ≥ 50 ELA procedures to ensure the standardization of ELA operation. The chief surgeon participating in this study has performed at least 50 PAD cases/year and accumulated ≥ 10 cases of ELA. Secondly, participant’s safety will be monitored in real-time by a combination of surveillance of EDC system and clinical monitoring.

The main purpose of this trial is to demonstrate the efficacy of ELA in de novo atherosclerotic lesions in LEAD compared with PTA, especially in popliteal and BTK arteries. The concept of “leave nothing behind” means more in the P2 segment. The laser could ablate the calcification of the vessel and the hyperplastic intima to reduce the occurrence of flow-limiting dissection after PTA. Then the stent implantation, which shows poor short- to long-term patency in P2 segment, could more likely be avoided. BTK artery is another challenge of endovascular treatment. Lesions involves BTK arteries are usually severely calcified and diffused and have a high incidence of restenosis after PTA [21]. Moreover, these patients have a higher proportion of diabetic mellitus or chronic kidney insufficiency, which makes them have a lower threshold of tolerance to surgery. Although minimal invasive, repeated endovascular procedures are impracticable. The longer working distance and smaller diameter of the catheter show good compatibility with BTK lesions and could significantly improve the patency.

The ELABORATE has limitations need to be demonstrated. Participants and surgeons are not blinded due to the real-world nature, which induces some selection bias cannot be precluded. Also, due to the real-world setting of the study, the lost follow-up rate is inevitably higher than RCT, and we enrolled more than 25% of the sample size to ensure the valid results.

### Current trial status

This trial has been registered in the Chinese Clinical Trial (ChiCTR2100051263) on September 17, 2021 after approval of the Ethics Community of Zhongshan Hospital, Fudan University (B2021-578) on August 30, 2021. The inclusion began on October 1, 2021, and 2 patients in the ELA group and 2 patients in the controlled group have been included. The other two centers are prepared to begin or are awaiting approval of the Ethics Committee. Inclusion will be continued until 2024, and the follow-up will be finished till 2024. The current trial protocol version is 1.0, and the informed consent version is 2.0.

ELABORATE is a multicenter, prospective study aiming at evaluating the safety and effectiveness of ELA combined with DCB in the treatment of de novo atherosclerotic lesions in a real-world setting. Data from ELABORATE will provide evidence for the efficacy of ELA in ASO. Meanwhile, the trial will make a reference for future evaluation of effectiveness of new therapies.

## Data Availability

Data can be provided to researchers upon request, subject to a review of privacy. Requests for data can be sent to the corresponding author by email.
